# Youth and family involvement in the development of a plain language trial results communication tool: CommuniKIDS

**DOI:** 10.1186/s40900-023-00499-2

**Published:** 2023-09-30

**Authors:** Ami Baba, Dawn P. Richards, Maureen Smith, Nicole Pallone, Shelley Vanderhout, Matthew Prebeg, Ellen B. M. Elsman, Beth K. Potter, Martin Offringa, Nancy J. Butcher

**Affiliations:** 1 Child Health Evaluative Sciences, The Hospital for Sick Children Research Institute, Toronto, ON Canada; 2 Clinical Trials Ontario, Toronto, ON Canada; 3 Canadian Organization for Rare Disorders, Ottawa, ON Canada; 4 CanPKU and Parent of a Child with an Inherited Metabolic Disease, Sparwood, BC Canada; 5 Clinical Epidemiology Program, Ottawa Hospital Research Institute, Ottawa, ON Canada; 6 School of Epidemiology and Public Health, University of Ottawa, Ottawa, ON Canada; 7 Department of Psychiatry, The Hospital for Sick Children, Toronto, ON Canada; 8 Institute of Health Policy, Management and Evaluation, University of Toronto, Toronto, ON Canada; 9 Division of Neonatology, The Hospital for Sick Children, Toronto, ON Canada; 10 Department of Psychiatry, University of Toronto, Toronto, ON Canada; 11 Peter Gilgan Centre for Research and Learning, The Hospital for Sick Children, 686 Bay Street, Toronto, ON M5G 0A4 Canada

**Keywords:** Patient and public involvement (PPI), Youth engagement, Family engagement, Patient engagement, Clinical trials, Plain language summary, Trial results

## Abstract

**Background:**

Pediatric trials are possible through voluntary participation of children, youth (age ≤ 18 years), and their families. Despite important arguments for trialists to provide trial progress or results, and evidence that participants desire it, this information remains rarely shared with youth and their families. Little guidance exists on how trialists can best communicate trial results back to participants and their families. Guided by Liabo et al.’s framework, we describe how we developed a pediatric-specific, “plain language summary” clinical trial results template called CommuniKIDS with an adult patient partner, family partner (parent), youth advisors, and parent advisors, taking into account their unique knowledge needs and preferences.

**Main text:**

Patient and Public Involvement (PPI) was integrated in the development of the CommuniKIDS template. In collaboration with Clinical Trials Ontario, we used a generic trial results template as a starting point. The core project leadership team included a patient partner and a family partner from project inception to completion. Five youth (ages 13–18 years) and eight parent advisors were consulted at each point of the development process through three virtual workshops conducted separately; youth workshops were led by a youth facilitator. During these workshops, advisors agreed on the importance and value of sharing trial results, and expressed their preferences on content, format, and timing of sharing trial results. PPI-led improvements included the addition of three new sections to the CommuniKIDS template: “at a glance,” “side effects,” and “next steps.” We reflect on our PPI strategy in the context of five “values” and six “practicalities” identified as good PPI principles, and summarize lessons learned when collaborating with youth and families from this project.

**Conclusion:**

Involvement of a patient partner, a family partner, youth advisors, and parent advisors in the development of CommuniKIDS was critical to create a clinical trial results template that is useful and relevant to its end-users. To our knowledge, CommuniKIDS is the first to meaningfully engage youth and parents as advisors and partners in developing a plain language summary results template for pediatric trial participants and their families. Our experience of co-developing CommuniKIDS demonstrates that meaningful PPI can be achieved in trial results communication and knowledge translation practices. This report provides resources for those seeking to involve youth and families in their initiatives and in meaningfully sharing trial results.

**Supplementary Information:**

The online version contains supplementary material available at 10.1186/s40900-023-00499-2.

## Abstract (plain language summary)

The voluntary participation of youth aged 18 and under in clinical trials makes it possible for researchers and healthcare providers to study medications and other treatments. However, most youth and their families who take part in clinical trials do not get any information on the trial’s progress or results, leaving many to wonder if anything useful came from their participation. There is an ethical obligation to give this information back to youth and their families, who might take risks by participating in trials. The aim of the CommuniKIDS project was to develop a “plain language summary” results template to share trial results back to youth and their families. Working with a patient partner, a family partner, five youth advisors (ages 13–18), and eight parent advisors, we set out to understand what youth and parents would like to see in a plain language summary of clinical trial results. The needs and preferences discussed with the advisors were included to create a child/youth health-specific template. The CommuniKIDS project is the first to involve youth and parents as advisors in developing a plain language summary results template for child/youth health trials. Here, we describe how we involved youth and parents in the development of CommuniKIDS, how the template was customized to be youth and family-friendly and reflect on lessons learned.

**Supplementary Information:**

The online version contains supplementary material available at 10.1186/s40900-023-00499-2.

## Background

Sharing trial results with clinical trial participants is good practice and ethical, as it fosters transparency and dissemination of knowledge to the people who helped generate it [1–5]. From the perspective of trial participants, it is a way for trialists to show appreciation, and may increase their willingness to stay enrolled in trials and participate in future research [5–7]. Not sharing trial results could be detrimental for trialists as patients have expressed unwillingness to participate in future research unless they were informed about the trial results [8]. Despite the importance of sharing trial results with participants, youth who participate in clinical trials, and their families, rarely receive information about trial results that are made possible with their participation: only 38% of 39 parents with children who participated in a study received a summary of the trial results, though 64% of 500 parents expressed a desire in receiving a summary of trial results should their child participate in a trial in the future [9, 10].

Clinical Trials Ontario (CTO) developed a plain language summary results template (“CTO template”) in collaboration with the clinical trials community as part of their Participant Experience Toolkit in 2021 [11]. While the development of the CTO template involved key knowledge producers and users, including patients and the public, this template was not designed specifically for pediatric trials. Youth and family caregivers have unique knowledge needs and preferences on how to receive trial results that can differ from those of adults; therefore, need to be involved in the development of guidelines and best practices on how trialists can share pediatric trial results [10]. Two recent studies involving youth and family caregivers explored the type of information, format, and layout that are preferred by youth and their families when receiving lay summaries of trial results [10, 12]. Preferences included a lay summary format that outlines study objective, methods, results, and details regarding side effects and effectiveness of the medication [12]. Another study found that youth ages 12–19 preferred receiving trial results in video format; for trial results shared in a written report, they preferred the use of infographics [10]. Additionally, the Good Lay Summary Practice guidance, adopted by the European Commission’s Clinical Trials Expert Group, includes different factors and recommendations to consider when preparing a lay summary for pediatric trial results [13]. In 2022, the updated European Union Clinical Trials Regulation mandated the publication of plain language lay summaries six months after completion of a pediatric clinical trial [14, 15]. Yet, there is a paucity of pediatric trial-specific guidelines, and plain language summary results templates co-developed with youth and family caregivers are still non-existent.

In this paper, we describe how we meaningfully implemented patient and public involvement (PPI) in the development of CommuniKIDS, a plain language summary trial results communication template for pediatric trials. We reflect on our PPI strategy in the context of Liabo et al.’s framework, which synthesizes principles essential to meaningful and positive involvement of the public [16]. We conclude with reflections on lessons learned while engaging youth and family caregivers in developing CommuniKIDS.

## Main text

### Overview of CommuniKIDS

In brief, the aim of this project was to co-develop a plain language summary clinical trial results template with youth and families by identifying the unique informational needs and preferences of this population [17]. We implemented an integrated knowledge translation approach [18–20] where various knowledge users (e.g., patient partner, family partner, youth advisors, and family advisors) were actively involved in the project from beginning to end. The final deliverable was a free and bilingual (English and French) template, along with a “user tip sheet” and an example of a completed template from a published pediatric trial, which can be found here: https://www.ctontario.ca/patients-public/resources-for-engaging-patients/toolkit-to-improve-clinical-trial-participants-experiences/plain-language-result-summary-for-pediatric-clinical-trials/

### Framework of youth and family involvement

We used Liabo et al.’s framework to guide the CommuniKIDS’ PPI strategy [16]. This framework synthesizes good practice principles for meaningful, positive involvement of the public in research [16], derived from a structured literature review. Liabo et al. identifies five “values”, which includes valuing different kinds of knowledge, inclusivity, partnership, transparency, and purposeful involvement [16]. Six “practicalities”, defined as approaches that enable the values, includes support, capacity building, proactive communication, proportional involvement, involvement throughout the project, and evaluation [16]. Below, we reflect on how these were implemented throughout the CommuniKIDS project; a summary of our PPI strategy in the context of the framework of involvement is in Table 1. Definitions of the five “values” and six “practicalities” are provided in Additional file 1.

**Table 1:** Summary of approaches (“practicalities”) that enable alignment with values in the framework of involvement [16]

Approaches (“Practicalities”)	Patient partner, family partner, youth advisors, and parent advisors’ involvement in the development of CommuniKIDS	Values
Support	• *Location*: All project team meetings and workshops were held through Zoom, enabling advisors from across Canada to contribute from their own homes without the cost, time, and potential health risks associated with travel and in-person meetings • *Compensation*: PPI honoraria was planned from the grant application stage. The patient partner, family partner, youth advisors, and parent advisors were all provided honoraria for their time and contributions according to the CIHR SPOR guidelines [22], and were offered reimbursement of childcare expenses if needed • *Dedicated staff*: The core project leadership team consisted of researchers, healthcare professionals, a patient partner, and a family partner. Both the patient partner and family partner led the development of the engagement strategy for youth and parent advisors. The project manager was responsible for communicating with team members and youth and parent advisors throughout the project. A dedicated youth facilitator was brought on to lead the youth workshops. At least one patient partner was present as a facilitator at the workshops	• Inclusivity • Partnership • Value different kinds of knowledge
Proportional Involvement	• Core project leadership team members decided on the frequency of project team meetings, which were held bi-weekly throughout the duration of the project • Youth and parent workshops were incorporated at key points of the project. At the first workshop, a high-level overview of when these workshops will take place during the project were discussed and agreed upon. Specific dates and times for workshops were coordinated based on the availability provided by the advisors	• Inclusivity • Partnership • Purposeful involvement • Value different kinds of knowledge
Capacity building	• The first workshop consisted of an explanation of the project purpose, what advisors would do during the workshops, and how advisors will be involved throughout the project. E-mail correspondence prior to each workshop included a summary of what advisors could expect to discuss at the workshop. To accommodate for different engagement styles, parent advisors were provided materials ahead of the workshop through e-mail to review if they wanted to • Creation of populated results summary and youth and parent-specific materials for workshop • Some members of the research team completed a Family Engagement in Research (FER) course • Two post-doctoral research fellows were part of the team, gaining valuable experience in PPI	• Partnership • Purposeful • Transparency • Value different kinds of knowledge
Proactive communication	• All meetings and workshops were held over Zoom. Communication between meetings and workshops were conducted through e-mail. Meeting agenda and minutes were shared after each meeting. Document sharing and editing was done through Microsoft OneDrive and e-mail • Involvement of youth and parent advisors in decisions related to major updates and improvements of the template • During workshops youth and parent advisors were encouraged to contribute through a communication method that best suited their needs, including using the chat, unmuting their microphone, and turning their camera on/off	• Partnership • Transparency
Involvement throughout the research	• The patient partner and family partner were involved throughout the entire duration of the project from the beginning to the end, led the development of the engagement strategy of youth and parent advisors, and contributed to the development of CommuniKIDS • Youth and parent advisors were engaged at three key points of the project to lend their expertise and perspectives in the development of the content and format of CommuniKIDS	• Inclusivity • Partnership •Value different kinds of knowledge • Purposeful involvement
Evaluation	• Youth and parent advisors anonymously completed a short, anonymous feedback survey adapted from the Public and Patient Engagement Evaluation Tool (PPEET) [24] • Feedback received was reviewed by core project leadership team members to inform future workshop planning and to reflect on lessons learned	• Inclusivity • Partnership • Transparency • Value different kinds of knowledge

### Practicalities (“approaches”) implemented in developing CommuniKIDS

#### Support to the patient partner, family partner, youth advisors, and parent advisors

The core CommuniKIDS project team was a diverse, multistakeholder team comprised of pediatric researchers, healthcare professionals, a patient partner with lived experiences of being a part of a clinical trial, a parent (family partner) of a child who participated in a clinical trial, and/or individuals with direct work experience with youth and families. The project team included team members from INFORM RARE [21], a Canadian research network focused on improving healthcare and outcomes for childhood rare diseases, and CTO, a non-profit organization actively involved in improving clinical trial conduct in Ontario and beyond. INFORM RARE and CTO team members were instrumental in the development of CommuniKIDS with regards to the PPI strategy, project materials, and dissemination of the final template.

**Fig. 1:**
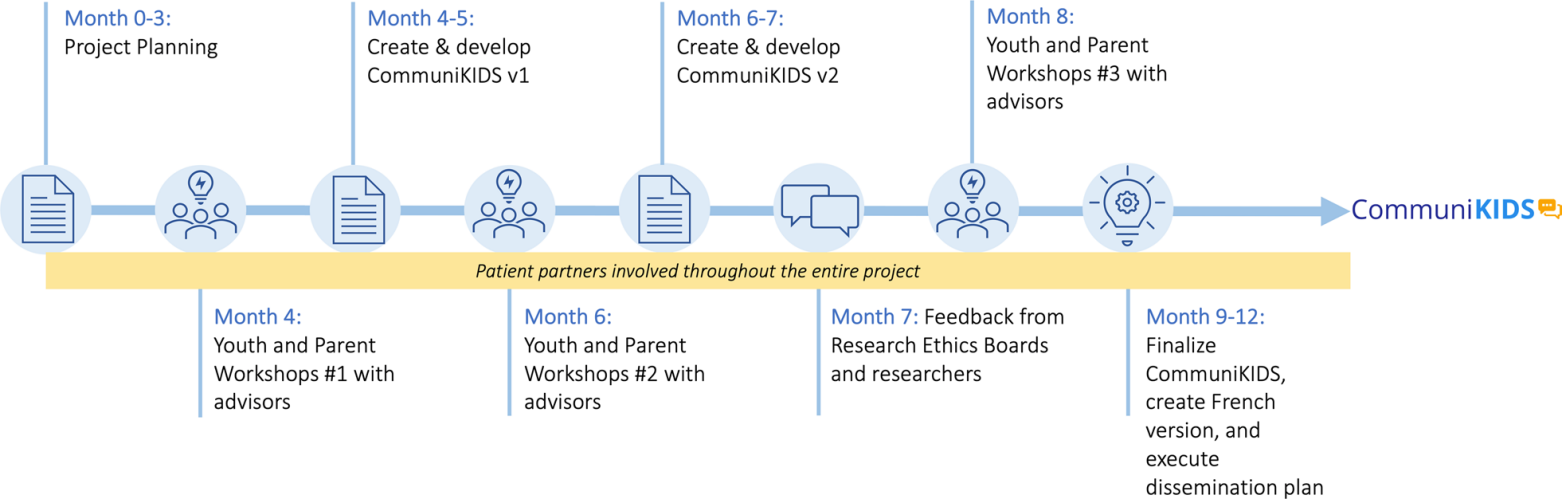
CommuniKIDS development process

As part of the core project leadership team, an experienced patient partner (MS) and family partner (NP) were involved in the project from the grant application stage to project completion and co-led the youth and parent advisors’ engagement strategy (Fig. 1). The patient partner is a citizen leader with lived experience as a patient with a rare disease diagnosis in childhood, and with experience in patient/citizen engagement in research. The family partner is a parent of a child diagnosed with Phenylketonuria (PKU), and has experience with family engagement in research, with a strong service history with the Canadian PKU and Allied Disorders Inc. patient advocacy groups as a vice president, board member, and volunteer. Another leadership team member (DPR) identifies as a patient with lived experience and contributed expertise from her role at CTO in engaging its community in the development of the CTO template. We engaged a graphic designer (MP) with expertise in youth engagement and knowledge mobilization to facilitate active stakeholder involvement within the design process itself to ensure that the design preferences of youth and parent advisors were reflected in the final CommuniKIDS template.

To engage youth and parent advisors, the CommuniKIDS team reached out by e-mail to several Canadian patient advisory groups with whom they were connected, including those related to pediatric rare diseases (INFORM RARE (https://www.informrare.ca/), CanPKU (https://canpku.org/), CureSMA (https://www.curesma.org/)), research networks (TARGet Kids! Parent And Clinician Team (https://www.targetkids.ca/pact)), and hospital networks (The Hospital for Sick Children (SickKids) Research Family Advisory Network and SickKids Children’s Council (https://www.sickkids.ca/en/careers-volunteer/volunteering/family-advisory-network/), Children’s Hospital of Eastern Ontario (https://www.cheo.on.ca/en/index.aspx), and Trillium Health (https://www.thp.ca/Pages/Home.aspx)). All interested individuals were invited to join. In total, five youth advisors (ages 13–18) and eight parent advisors joined CommuniKIDS with diverse research and healthcare experiences, including youth with lived experience as a participant in clinical trials or other clinical research, and parents to youth who have participated in clinical trials or other research. Youth and parents involved with CommuniKIDS were not related and were not from the same household. Half of the parent advisors disclosed that they or their child have participated in a clinical trial or clinical research study before. Of those that disclosed their location, parent advisors were located throughout Canada in Ontario (n = 3), New Brunswick (n = 2), and Quebec (n = 1); youth advisors were in Ontario (n = 3) and British Columbia (n = 2).

At least four core project leadership team members attended each virtual advisory workshop; they took on the role of co-facilitators, notetaker, and technical support. Each parent workshop had at least two facilitators, one being a researcher (NJB) and the other a family partner (NP). To create an environment where youth advisors felt comfortable to share their thoughts, an experienced young adult youth facilitator led the youth workshops. All youth and parent workshops were held separately. To schedule workshops, a Doodle poll was used. We offered core project team members and advisors different possible dates and times to accommodate for group preferences, including outside of business hours, such as weekday evenings and weekends. Based on preferences garnered from all advisors and core project team members in the Doodle poll, all workshops were scheduled on Saturdays.

Honoraria to recognize the contributions of the patient partner, family partner, youth advisors, and parent advisors were planned and dispersed based on the Canadian Institutes of Health Research (CIHR) Strategy for Patient Oriented Research (SPOR) guidelines of $25 CAD/hour [22] via cheque or gift cards, based on their preferences. Due to the ongoing global COVID-19 pandemic that restricted the ability for in-person meetings and workshops, all core project leadership team meetings and advisor workshops were held over Zoom. This also allowed us to engage with project team members and advisors across Canada, which turned out to be beneficial to the advisors’ involvement and input. It provided the flexibility to involve team members, youth advisors, and parent advisors without geographical restrictions, and reduced burden on youth advisors and parent advisors as they did not have to travel to a (distant) physical location to attend workshops. This could be especially relevant to youth living with medical conditions impacting their ability to travel, and parents who care for children with medical conditions.

As patient and public partners may have unique priorities and goals for being involved in academic work, we decided on an approach where advisors were first asked to opt in or out of being named on project materials, and to indicate at what level they wished their contribution to be recognized. Parent advisors were given the option of being acknowledged by their full name, their initials, or as an anonymous contributor. Youth advisors were given the option of being acknowledged by their first name, or as an anonymous contributor, that is, as part of the “CommuniKIDS Youth Advisors.” Additionally, for youth who were part of the INFORM-RARE Youth Advisory Group, we reached out to their parents to ask for their preferences in accordance with the INFORM-RARE Youth Advisory Group policies. These options were discussed with all advisors at the final workshop. We asked all advisors to indicate their preferences after they had time to think about the different options presented.

#### Proportional involvement

The core project leadership team set the frequency of project team meetings, which was bi-weekly throughout the duration of the project. Our engagement strategy for youth and parent advisors were to engage and consult them at key points of the project during three virtual weekend workshops between November 2021 and April 2022 (Fig. 1). Details regarding youth and parent advisors’ involvement in each workshop are below.

#### Capacity building

We conducted youth and parent workshops separately to ensure all workshop contributors felt comfortable, and to prevent any power imbalances between youth and adults during the workshops. To provide youth and parent advisors a visual example of what the template could look like when completed and in use, the CTO template was populated with results from a published clinical trial to create a populated results summary to discuss during the workshops. Results from a pediatric migraine trial [23] was used in this populated results summary, as migraines are a common condition broadly known and understandable to youth and families. Additionally, this trial was stopped early due to futility which allowed the project team and the advisors to explore how these types of results could be communicated in a trial results communication tool.

Prior to each workshop, the core project leadership team prepared workshop materials to support the review of the populated results summary, which included slide decks, probing questions, and interactive activities such as icebreakers, polls, and word clouds, customized specifically for youth and parent workshops. Before the first parent workshop, a “mock” workshop was held to rehearse the workshop in its entirety with “mock workshop attendees”, which comprised of leadership team members and other invited colleagues who were not involved with CommuniKIDS. This “mock” workshop resulted in slight adjustments of the materials. For subsequent workshops, facilitators met before the workshops to assign slides and go over talking points. Parent advisors were sent materials, such as the populated results summary, in advance of each workshop. We communicated to the parent advisors that materials were sent in advance to give them the opportunity to review should they wish to but emphasized that there was no expectation to do so. Through consultations with the patient partner, family partner, and youth facilitator, we elected to not send youth materials prior to the workshop.

As the workshops progressed, the populated results summary was modified iteratively based on elicited preferences of the youth and parent advisors during the workshops, and it eventually evolved into an example completion of the final CommuniKIDS template for summarizing pediatric trial results (https://www.ctontario.ca/cms/wp-content/uploads/2022/05/Populated-CommuniKIDS-Template-May-2022-Final-002.pdf). This example and its contents were reviewed and approved by the trial authors through e-mail [23].

#### Proactive communication

During the workshops, we encouraged advisors to use the Chat function on Zoom, unmute their microphone to ask questions or share their thoughts, and left it to each advisors’ discretion to leave their camera on or off with recognition of everyone’s comfort levels. In between workshops, the project manager (AB) communicated with the youth and parent advisors through e-mail; correspondence centered around details about workshops, scheduling of workshops, arranging honoraria, and providing project updates.

#### Involvement throughout the research

A series of three virtual workshops were conducted; however, as youth and parent workshops were all conducted separately, there were a total of six virtual workshops. Below, we describe how advisors were involved in the series of virtual workshops:

*Workshop #1* The objective of the first workshop was to introduce advisors to the purpose and value of CommuniKIDS, set expectations for the advisors’ involvement, and explore the value of a pediatric specific trial results communication tool. Advisors were given the opportunity to ask questions about the proposed template and the project objectives. At the first youth workshop, we worked with the youth advisors to co-create ground rules to foster safe and productive workshop discussions.

Discussions focused on the first version of the populated results summary from the example trial, which served as a starting point to explore the unique knowledge needs and preferences of youth and their families in a trial results communication template. We also discussed lay-friendly language and terminology, level of detail for each section of the summary, and formatting of various types of information such as location and numbers. Youth and parent advisors provided feedback and comments on each section, including its purpose and importance. They were also encouraged to make suggestions for new sections of content that were not present on the populated results summary.

After the first workshop, feedback from both the youth and parent workshops was reviewed by the core project leadership team and feedback was integrated to make changes to the populated results summary prior to workshop #2. In addition to revising the populated results summary, the core project leadership team created a blank template as the first draft of our CommuniKIDS template. This blank template only contained instructions for template users (e.g., researchers) on what each section should contain.

*Workshop #2* The updated populated results summary was presented to advisors for review. The parent workshop also discussed the blank template that contained the instructions. Discussions in workshop #2 focused mostly on the content (level of detail, word choice, and understandability) and formatting (layout, spacing, colours used) of the blank template and populated results summary.

The CommuniKIDS core team revised the blank template and populated results summary based on the feedback received from the advisors again. The graphic designer (MP) helped address design issues discussed in workshop #2, such as streamlining the template design and ensuring accessibility. The CommuniKIDS team also developed a CommuniKIDS User Tip Sheet (https://www.ctontario.ca/cms/wp-content/uploads/2022/05/CommuniKIDS-Considerations-Tipsheet-May-2022-Final.pdf) to serve as a resource for template users beyond the instructions in the blank template.

*Workshop #3* In workshop #3, we presented both the revised blank template and populated results summary to the parent advisors along with the CommuniKIDS User Tip Sheet. With the youth advisors, we discussed the revised populated results summary and CommuniKIDS User Tip Sheet. The “instructions for researchers” was not reviewed with youth based on advice of the patient partner, family partner, and the youth facilitator to avoid “overloading” the session, and to allow the youth sufficient time to discuss the populated summary and user tip sheet*.* Prior to meeting the advisors for workshop #3, we sought feedback on the template and associated materials from various stakeholders, which included trialists, clinical research professionals, and research ethics board (REB) members. We sought feedback from stakeholders on (1) whether the CommuniKIDS template is suitable and practical for trialists to use in communicating trial results to youth and family caregivers, and (2) whether they had any concerns from a research ethics perspective in communicating trial results using this template. Feedback from the 24 stakeholders were reviewed with the advisors. Potential modifications to be made to the template based on stakeholder feedback were discussed, such as rephrasing key terms (e.g., “study/trial limitations”), and the addition of a section dedicated to detailing patient and family engagement in the trial. Advisors provided final rewording and formatting suggestions. The workshop concluded with a roundtable discussion on the value and importance of sharing trial results back to trial participants, the appropriate time to share trial results with youth and parents, and the importance of having the option to opt in and out of communications from the trial team.

#### Evaluation

After each workshop, we asked advisors to complete a short, anonymous feedback survey adapted from the Public and Patient Engagement Evaluation Tool (PPEET) [24]. Survey feedback helped in planning subsequent workshops and allowed the team to reflect and identify what went well and what could be improved. Advisors commended the organization of the workshops and the facilitators, and provided suggestions on how to ensure all voices were heard during the workshops. This included keeping workshop groups small and to create an environment and opportunity where each person can feel that they can speak, such as through a roundtable approach or breakout rooms. When utilizing a virtual format, having a designated person read comments and watch for “hands raised” during the virtual meeting was thought to be helpful in increasing participation and input from attendees.

Overall, the success of our engagement strategy is evident from the overwhelmingly positive feedback from the advisors after each workshop, with the majority of advisors responding to questions such as “I was satisfied with today’s workshop” with “Strongly Agree” or “Agree”. Feedback surveys highlighted that parent advisors appreciated the communication, organization, and open discussions during the workshops. Youth advisors shared very positive feedback about the workshops and felt able to share their views freely and that they learned more about communicating clinical trial results. Encouragingly, all youth advisors expressed a desire to contribute towards other projects in the future where they could collaborate with researchers, with one youth leaving the following comment in the last feedback survey: “this was an amazing experience to have and loved every minute of it.”

### Workshop findings: recommendations for sharing trial results and final CommuniKIDS template

The meaningful engagement of the patient partner, family partner, youth advisors, and parent advisors led to multiple iterations of the template, with improvements made to the template content and design that reflect the unique knowledge needs and preferences of youth and their families. Although youth and parent workshops were conducted separately, there were many similarities in the feedback received. Not part of the CTO template, youths’ specific suggestions included beginning the template with a “Thank you” section to ensure that gratitude is communicated as a primary message, in addition to having an executive summary of the trial at the beginning of the template. Youth also placed heavy emphasis on how risks associated with side effects and benefits of the intervention can be communicated.

The final CommuniKIDS template features various PPI-led improvements: the addition of three new sections not featured on the CTO template, including (1) an executive summary of the trial (“At A Glance”); (2) a dedicated section for side effects, with suggestions on how to quantify their frequency and severity (“Side Effects”); and (3) a section where trialists can elaborate on next steps of the trial (e.g., publications, future phases of the trial), and include pointers on how youth and their families can discuss trial results with their doctor (“Next Steps”). All PPI-led improvements made to the template are summarized in Table 2.

**Table 2:** PPI-driven improvements made to the CommuniKIDS template

Template section	Improvements
Thank you	Template begins, rather than ends, with Thank You section
At a glance^a^	Provides an executive summary of trial to template readers
	Subheadings are hyperlinked to the corresponding main section in the template for ease of navigation
Trial information	Combines “Trial Information” and “For more information” section
About the trial	Contains four subsections phrased as question prompts to provide overview of trial conduct
	Addition of section dedicated to elaborating on patient and family engagement in trial Coverage of trial phases (phase 1–4)
Participants	Addition of section to discuss inclusion/exclusion criteria Recommendation to consider PROGRESS-Plus points [39]
	Simplified way to represent participant numbers (e.g., those who started, randomized to treatment, completed the trial)
	Recommendation to use graphics to demonstrate participant numbers
Location	Option to add written description along with a visual representation of location (e.g., map)
Findings	Use table(s), graphic(s), numbers, and percentages
	Distinguish patient reported outcomes if relevant
	Use reader friendly terminology
Side effects^a^	Addition of standalone side effects section, along with an example side effects table
	Quantify frequency and severity of side effects
Trial limitations	No changes made here
Next steps^a^	Addition of section to elaborate on trial next steps (e.g., next phase of trial, publications)
	“Discussing This Trial with your Doctor” section moved here
	Recommendation to insert infographic if relevant
Changes not specific to a section	Template design and formatting improved
	User tip sheet created for template users, divided into four sections for template users’ consideration

^a^New section added to template based on PPI feedback

The CommuniKIDS template can be found here (https://www.ctontario.ca/patients-public/resources-for-engaging-patients/toolkit-to-improve-clinical-trial-participants-experiences/plain-language-result-summary-for-pediatric-clinical-trials/) and is included as Additional file 2. In CTO’s Participant Experience Toolkit [17], template users can choose from two editable formats (Canva and Microsoft Word) along with the CommuniKIDS User Tip Sheet. As a resource, the final revised populated results summary from the Powers et al. (2017) trial [23] used during the workshops is available as an example. The template and the user tip sheet are available in English and French. The template design was updated to be compliant with the Accessibility for Ontarians with Disabilities Act (AODA) guidelines [25] to improve the accessibility, presentation, readability, and appeal of the template for youth. Colours and icons within the template can be customized to an organization’s colour scheme, and in accordance with the needs of the trial.

CommuniKIDS advisors recommended researchers share trial results back to youth and their families so that those who participated in trials can understand the trial’s implications and impact, see if the trial findings affect them, and decide whether they can act on the results or talk to their doctor. Youth and parents shared that they preferred to receive trial results directly by e-mail over other online methods or postal mail. The importance of choice was emphasized in receiving trial results—while parents and youth felt that by default, anyone who signs up for a trial should receive trial results, they recognized that some may not want to receive correspondence; therefore, having the ability to opt in or out of receiving any information on the trial was considered important. For trials ongoing for over a year, sharing periodic updates with a final summary of results at the end was preferred, rather than receiving everything at the end.

## Discussion

We have co-developed a pediatric trial results communication template with a patient partner, family partner, youth advisors, and parent advisors. Our experiences highlight the value in working with patient partners, youth, and parent advisors when designing a tool to convey trial results to youth and their families.

The final version of the CommuniKIDS template reflects preferences consistent with other studies that gauged pediatric and family caregiver preferences for sharing clinical trial results back to the pediatric population. Fernandez et al. found that adolescents and parents wanted to know the reasons for the study, next steps for researchers, and what the results were, including positive and negative results, amongst others [2]. Zimmerman et al. found that youth and family caregivers preferred a formatted lay summary that clearly goes over the purpose of the study, methods, and results, including medication effectiveness and information on side effects [12]. Chakraborty et al. found that while video was the most preferred format for youth ages 12–19, other preferences included a written report in a digital format that integrated infographics [10]. The final CommuniKIDS template is a plain language summary results template that is formatted and has clear sections, and encourages the use of infographics, such as in the “Participants”, “Location”, “Results”, and “Next Steps” sections. While past work has focused mostly on surveying or interviewing youth and/or families for their preferences, or feedback on various plain language summaries [2, 10, 12, 26], CommuniKIDS is the first, to our knowledge, to engage youth and parents as advisors and partners in developing a plain language summary results template for youth, through the implementation of approaches and alignment with values that define meaningful involvement.

The use of CommuniKIDS should be applied and modified as appropriate in partnership with the specific patient groups who will be receiving the lay summaries. Additionally, in communicating trial results, researchers need to appropriately plan and consider various resources early in the research planning process to foster meaningful PPI, account for associated costs, and remain flexible throughout the project [5, 27]. This includes budgeting for activities associated with sharing trial results and working with pediatric research partners on a “study results sharing plan” to determine timing of sharing results, mode of communication, and the preparation of materials using plain language [5, 27].

In the future, we also seek to collect and evaluate experiences of pediatric trial teams that utilize the CommuniKIDS template.

### Framework of involvement: reflections on approaches and alignment with values in the co-development of CommuniKIDS

We used approaches (“Practicalities”, Additional File 1) to enable and align our co-development of CommuniKIDS with the values outlined in Liabo et al.’s framework of involvement (Table 1), described below.

#### Value different kinds of knowledge

Partnering with a patient partner and family partner with extensive experience in family, youth, and patient engagement methods throughout the entire project allowed development and implementation of an effective engagement strategy to work with youth and parent advisors at strategic points. This included accommodating for different preferences such as reviewing material prior to the workshops and creating youth and parent-specific materials for the workshops. Youth and parent advisors contributed their lived experiences to ensure that the final template is relevant and usable for other youth and families who may want trial results after clinical trial participation. Fostering an environment where different perspectives were valued allowed for mutual learning to occur between the patient partner, family partner, advisors, and researchers [28].

#### Inclusivity

A diverse range of advisors was engaged from across Canada. Some had specific clinical trial experience, while others did not; everyone’s feedback was valued and discussed for integration into the final version of the template. Additionally, we had a patient partner or family partner as a facilitator at each workshop, and a dedicated youth facilitator for the youth workshops, in the hopes that this would foster an environment where advisors felt comfortable in asking questions and sharing their feedback, regardless of their experience in partnering with researchers. Having an individual with lived experiences relatable to patient and public partners has shown to be effective in managing power imbalances between partners and researchers [29]. Honorarium for advisors ($40 per 1.5-h workshop), the patient partner, and family partner were offered to recognize their time and contributions, and we took their preferences of compensation method into consideration; this allowed for people who would not or could not contribute otherwise to join. Additionally, integrating preferences and consulting public partners regarding compensation methods has been shown to be a practice appreciated by public partners in research [28].

#### Partnership

Respect between advisors and the core project leadership team, which comprised of a patient partner and a family partner, was maintained throughout the entire project to develop a trusted partnership. The core project leadership team itself comprised of many individuals with diverse backgrounds and range of perspectives from different organizations, demonstrating true partnership. We scheduled advisory workshops on weekends, when needed, to accommodate school, work, and family commitments. We also remained flexible with regards to attendance and welcomed them to join in when they were able to in a method that best suited them (e.g., Zoom chat, phoning in, comments by email).

#### Transparency

Regular communication was maintained to provide updates, reminders, and relevant materials to support the advisors to contribute to their full potential. Involvement in the project, and its purpose, was made clear from the beginning to all partners and advisors, resulting in commitment from partners and advisors to the project. Transparency was maintained by reviewing the changes and updates made due to their feedback during the workshop. Facilitators also took time at the beginning of each workshop to build rapport between the researchers and advisors, which is important in understanding the advisors’ intentions and interests in the project [30]. Open communication and maintaining transparency were critical to build mutual respect, trust, and a sense of togetherness [28].

#### Purposeful involvement

The purpose of involving a patient partner, family partner, youth advisors, and parent advisors was made clear at the beginning of the project and workshop series, and throughout the project. We emphasized how the perspectives of youth and parent advisors were integral in developing a useful and relevant plain language summary results template. In feedback surveys, youth and parent advisors both felt as though their suggestions from earlier workshops were integrated into each updated version of the template, including the final version. At the end of the project, all advisors felt that the CommuniKIDS workshops achieved their goals, according to the feedback survey results.

### Lessons learned

Below, we discuss lessons we learned around three aspects of involving youth and parents in our project that merit special attention: ethical considerations, practical facilitators and barriers to working with youth, and the importance of true project partnership.

#### Acknowledge ethical considerations surrounding affiliation to project

We were cognizant of the fact that many of the advisors had lived experiences as a clinical trial participant or being a parent to a child who participated in a clinical trial. We learned that the process of acknowledging, and potentially naming contributors, has many considerations that need to be weighed. As both youth and parent advisors were a part of our project, we had a responsibility to ensure their understanding of the full implications of being named on project materials, such as the possibility of others assuming or linking their association with the project to their health or involvement in clinical trials. In consideration of this, enough information on the possible implications of being named on materials, and the option to opt in or out of being acknowledged publicly should be offered to patient and public members.

#### Consider facilitators and barriers to youth engagement

Creating an open, inclusive, and safe environment is crucial when engaging youth [31]. Youth may feel intimidated to share their thoughts or feel outnumbered in an environment with adults [32–34]. Creating a group with other youth to have balanced representation may remove potential barriers to participation and the feeling of a power imbalance [32]. Designing materials, such as youth-specific slides and interactive activities is important to maximize their understanding and confidence in their ability to contribute. Working with youth to co-create ground rules will empower youth in thinking of ways to work respectfully with others and contribute productively [35]. Providing youth with a platform to be involved in change and share their thoughts or feedback will give them a sense of authority, confidence, and purpose [30, 36].

#### Importance of true project partnership

We learned and emphasize the importance and value of working with patient and public contributors. Recognizing the contributions of individuals beyond simply ‘patient’ or ‘public’ partner means recognizing one’s own unique lived experiences, education, training, and research and health care system experiences [37, 38]. True to the principle of valuing different kinds of knowledge, lived experience that patient partners and advisors bring must be considered expertise. Youth and family members must be engaged in using the CommuniKIDS template to create plain language summaries of pediatric trials. We urge research teams to consider individuals beyond their label as a ‘patient’ to fully engage them in a meaningful manner throughout a project.

### Limitations

As the CommuniKIDS youth advisors were all between the ages of 13–18 years, the CommuniKIDS template may not capture the content and format needs of youth under the age of 13. Further investigation is needed to explore unique needs of youth under the age of 13, as well as in the development of effective and appropriate engagement strategies while considering their capability and capacity. Although our recruitment strategies did include patient groups with developmental disability and rare disease, we did not collect medical information about the youth advisors. The views of youth with developmental disabilities and other specific pediatric populations may therefore not be adequately represented in this template. Moreover, while we had a patient partner and a family partner as part of the core project leadership team, and a youth facilitator who led the youth sessions, we did not have any youth partners. We recognize that there were more adult contributors compared to youth advisors, as we had fewer youth who signed up to contribute than parents despite similar recruitment efforts. However, as described, we implemented methods to foster a safe, welcoming workshop environment for our youth advisors to share their thoughts and feedback, such as through engaging a youth facilitator close to their age and—to avoid any dominance of adult contributors—conducting youth workshops separately from parent advisors. Additionally, as our advisors were all located in Canada, we may not have captured the needs and perspectives of those outside of Canada.

## Conclusions

CommuniKIDS is the first project to engage youth and parents as advisors and partners in developing a plain language summary results template for youth who participate in trials and their families, through the application of approaches aligning with values of meaningful involvement. The resulting contributions and feedback from the youth and parent advisors were essential to the development of a template that is relevant and useful to the pediatric population who are the end-users of trial results. Our reflections on our PPI engagement strategy and lessons learned can serve as a resource for future groups for how to work with youth and families. Future directions include collecting and evaluating experiences in using the CommuniKIDS template by pediatric trial teams.

### Supplementary Information

**Additional file 1.** Definition of values and practicalities in the framework of youth and parent advisor’s involvement, adapted from Liabo et al. [16].

**Additional file 2. **CommuniKIDS template.

## Data Availability

Data sharing is not applicable to this article: no datasets were generated or analyzed during this project. The final CommuniKIDS template is available as part of the Clinical Trial Ontario (CTO) Participant Experience Toolkit: https://www.ctontario.ca/patients-public/resources-for-engaging-patients/toolkit-to-improve-clinical-trial-participants-experiences/plain-language-result-summary-for-pediatric-clinical-trials/

## Abbreviations

CTO: Clinical Trials Ontario
PPI: Patient and public involvement
REB: Research ethics board
CIHR: Canadian Institutes of Health Research
SPOR: Strategy for Patient Oriented Research
PPEET: Public and Patient Engagement Evaluation Tool
AODA: Accessibility for Ontarians with Disabilities Act
