# Supplementary material for: Youth and family involvement in the development of a plain language trial results communication tool: CommuniKIDS
Source: Res Involv Engagem. 2023 Sep 30;9:88. doi: 10.1186/s40900-023-00499-2 (PMC10544151; doi:10.1186/s40900-023-00499-2)
Supplement: Supplementary file 1 — Additional file 1. Definition of values and practicalities in the framework of youth and parent advisor’s involvement, adapted from Liabo et al. [16]. [file 40900_2023_499_MOESM1_ESM.docx]

**Additional File 1.** Definition of values and practicalities in the framework of youth and parent advisor’s involvement, adapted from Liabo et al.^a^

| 1. **Values** | **Definition** |
| --- | --- |
| 1. Value different kinds of knowledge | Recognition of the expertise that youth and parent advisors can bring to the team to compliment what researchers bring to the table. |
| 1. Inclusivity | Opportunity for different individuals to be involved equally regardless of social backgrounds and abilities. |
| 1. Partnership | Researchers and involved youth and parent advisors working together on the team, being respectful for one another’s roles and contributions. |
| 1. Purposeful involvement | Clear rationale for everyone’s roles and involvement in the project, which is communicated to all team members, as well as commitment from all team members involved. |
| 1. Transparency | Honesty and openness between researchers and youth and parent advisors, and maintain open lines of communication between all team members on what is being done and how. |
| 1. **Practicalities** | **Definition** |
| 1. Support | Advocating for youth and parent advisors’ participation in team efforts and providing practical assistance to public members/advisors to enable their continued contributions to the team, such as through allocating funds to compensate for time, reimburse travel costs, and accounting for varying needs to make meetings and continuous participation accessible and possible. Having dedicated staff enabling advisors’ involvement and accommodating for different needs. |
| 1. Proportional involvement | Customizing involvement of youth and parent advisors in the research based on the needs of the project and in consideration of resources and other competing demands. |
| 1. Capacity building | Training for all team members (researchers and advisors), and co-learning between the two groups. |
| 1. Proactive communication | Ensuring youth and parent advisors are kept informed as the project progresses and ensuring that modes of communication are suitable for everyone. |
| 1. Involvement throughout the research | Opportunity for youth and parent advisors to be involved and contribute their expertise at any stage of the project in various ways and capacities. This would be made possible by having clear guidelines, framework, and leadership to allow for public involvement. |
| 1. Evaluation | Identification of good practices enabled through effective communication, co-learning, and research. |

^a^ Liabo K, Boddy K, Bortoli S, Irvine J, Boult H, Fredlund M, et al. Public involvement in health research: what does 'good' look like in practice? Res Involv Engagem. 2020;6:11.
