# Supplementary material for: Youth and family involvement in the development of a plain language trial results communication tool: CommuniKIDS
Source: Res Involv Engagem. 2023 Sep 30;9:88. doi: 10.1186/s40900-023-00499-2 (PMC10544151; doi:10.1186/s40900-023-00499-2)
Supplement: Supplementary file 2 — Additional file 2. CommuniKIDS template. [file 40900_2023_499_MOESM2_ESM.docx]

This is where the name of your trial goes

[If needed, this is where your subtitle or more information about your study goes (e.g., full study name).]

**Thank you!**

[Language to consider: Thank you for volunteering your time and effort to this clinical trial. (If the study is a long, ongoing study: We appreciate your commitment to this study over X number of years.) We sincerely appreciate your contribution to this trial and in helping to advance medical knowledge.]

# At a glance.

## [Our goal:](#_What_did_we)

[1-2 sentence(s) about the primary aim of the trial.]

## [Participants:](#_Number_of_participants:)

[A sentence describing who participated in the trial.]

## [Results:](#Results)

[1-2 sentence(s) about the key findings of the study.]

## [Side effects:](#Side_Effects)

[A sentence describing key side effects of the intervention.]

## [Next steps:](#_Discussing_this_trial)

[A sentence describing what the next steps or follow-up will look like.]


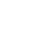


[Include the following information, as applicable: Trial name, investigator name(s), sponsor, funder, study start/completion dates, clinical trial identifier or registration/universal trial number.]

## For more information:

[If relevant, add in link(s) to any publication(s), study website and/or contact information for participants to request the publication if it’s not publicly available or to ask questions about the trial, e.g., whether they received a placebo or treatment.]

# About the trial.


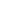


## What did we do?

[1-2 sentence(s) about what the study did to achieve its aim (e.g., we compared two medications and a placebo).]

## How did we do it?

[1-4 sentence(s) about the study's methods.]

## What did we want to know?

[1-2 sentence(s) about the study's hypotheses or research questions.]

## How did we engage patients/families?

[If relevant, add in 1-2 sentence(s) about how the trial engaged patients/families in the planning and conduct of trial, interpretation of results, and/or preparation of this summary.]

## Study phase:

Phase 1

Phase 2

**Phase 3**

Phase 4

[If relevant, consider including 1-2 sentence(s) to explain what the study's phase means (e.g., Phase 3: This phase focuses on whether the drug

works well and how safe it is). Shade in the appropriate phase for your trial. If study phase is not applicable to your trial, consider replacing section with trial type/design.]

Curious about what the other phases are? Learn more on the Clinical Trials Ontario webpage [here](https://www.ctontario.ca/patients-public/learn-more-about-clinical-trials/i-want-to-learn-about-clinical-trials/).


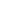

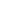

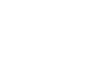


[List the main inclusion/exclusion criteria of the trial in bullet point form or provide a brief summary of who could participate in the trial. Consider including link to the full inclusion/exclusion trial for the trial. Consider applying [PROGRESS+](https://methods.cochrane.org/equity/projects/evidence-equity/progress-plus) when discussing trial participants.]

## Number of participants:

[Numbers overall and participants per arm. Add in definitions of technical terms (e.g., “randomized”). Differentiate between number of participants who started the trial/were randomized to treatment groups versus those that completed the trial, as appropriate.]

[Tip: use graphics to demonstrate number of participants (e.g., people graph, pie charts) for a visual representation.]

# Location.

[If needed, written description of the location(s) relevant to the trial.]

[Tip: use a map or list sites if it is a

multi-site trial.]

[This section may be broken down into subheadings (e.g., primary and additional secondary outcomes) described in neutral, unbiased language. If applicable, consider identifying patient-reported outcomes. When reporting results in relation to number of participants, include percentages as well as numbers. Differentiate between any short or long-term effects, as appropriate.]

[Consider noting that no individual results are provided in this document, and/or including information on the potential impact of the results on the population under study.]

[Tip: use tables and graphics (e.g., graphs and charts) to explain and show results.]

[Consider including a side effects section here, as applicable. Depending on your trial, consider renaming this section to best fit your purpose (e.g., psychological adverse effects). Include and focus on the most frequent, most serious, and/or unexpected side effects in this section, and provide reasoning behind listing the ones that are on the template.]

[A table describing the number and percentage of side effects in each treatment group may be beneficial – feel free to modify the table below as appropriate to fit your specific trial findings. Define or quantify what mild, moderate, and severe means in the context of your trial (e.g., as per [GCP guidelines](https://www.canada.ca/en/health-canada/services/drugs-health-products/drug-products/applications-submissions/guidance-documents/international-conference-harmonisation/efficacy/clinical-safety-data-management-definitions-standards-expedited-reporting-topic.html)). Consider including a link where individuals can access all side effects experienced during the trial for those who want further details.]

**EXAMPLE TABLE:**

| Side Effect | Frequency | | | | Severity | | | Long Term Impact | |
| --- | --- | --- | --- | --- | --- | --- | --- | --- | --- |
|  | Very likely (>75%) | Likely  (50-75%) | Less Likely  (5-50%) | Rare (<5%) | Mild | Moderate | Severe | Temporary | Permanent |
| Headache |  |  |  |  |  |  |  |  |  |
| Fatigue |  |  |  |  |  |  |  |  |  |
| Abdominal pain |  |  |  |  |  |  |  |  |  |
| Diarrhea |  |  |  |  |  |  |  |  |  |
| Irritability |  |  |  |  |  |  |  |  |  |
| Cough |  |  |  |  |  |  |  |  |  |
| Nasal congestion |  |  |  |  |  |  |  |  |  |
| Dizziness |  |  |  |  |  |  |  |  |  |

# Trial limitations.

**!**

[If applicable, this section may include information on what the trial couldn’t show, results for populations that were excluded, etc.]


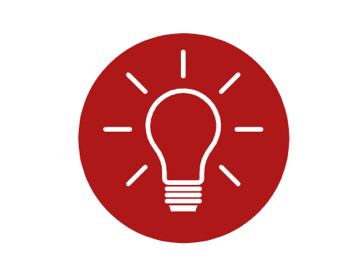


[Include details here on conclusions and interpretations drawn from the trial findings and results.]

**Next steps.**

[Include details here about what’s next for the research (e.g., moving to phase 3 after phase 2, another study, publication, licensing), information or other opportunities to be involved in future phases of the trial if applicable, what the findings of this trial mean for people who live with the condition studied (e.g., clinical care), and whether and when the intervention might be available after the study, etc.]

## Discussing this trial with your doctor:

[Consider inserting a summary infographic here. Language to consider for this section: Let your/your child’s doctor know the results of the trial that you/your child participated in, experience in participating in the trial (e.g., when the trial started/ended), and whether the trial intervention worked or did not work.]
